# Analysis of a simple disk diffusion method to evaluate ceftazidime–avibactam/aztreonam combination synergism against New Delhi metallo-beta-lactamase-producing clinical isolates

**DOI:** 10.1099/acmi.0.001049.v4

**Published:** 2025-12-15

**Authors:** Reena Rajan, Sasikala Gopinathan, A. V. Raghavendra Rao, Rajarajeswari B, Sureshkumar Mathavi

**Affiliations:** 1 Department of Microbiology, Vinayaka Mission’s Kirupananda Variyar Medical College & Hospitals, Vinayaka Mission’s Research Foundation (Deemed to be University), Salem-636308, TN, India; 2 Department of Microbiology, Annapoorana Medical College & Hospitals, Salem, TN, India; 3 Department of Microbiology, Vinayaka Mission’s Kirupananda Variyar Medical College & Hospitals, Salem, TN, India

**Keywords:** ceftazidime–avibactam/aztreonam, synergic combination, *in vitro* synergy, multiple carbapenemase producer, *Enterobacterales*, *Pseudomonas aeruginosa*

## Abstract

The rise of multidrug-resistant *Enterobacterales* and *Pseudomonas aeruginosa* has diminished the reliability of conventional antibiotics for treating Multidrug Resistant (MDR) infections. The combination of ceftazidime–avibactam with aztreonam has demonstrated *in vitro* synergism against multidrug-resistant organisms, notably metallo-beta-lactamase-producing strains. Treatment with the ceftazidime–avibactam/aztreonam combination may provide clinical benefits for patients with multidrug-resistant bacterial infections. The present study aimed to detect genes encoding carbapenem resistance in clinical strains and to determine the efficacy of ceftazidime–avibactam/aztreonam against carbapenemase co-producers. A cross-sectional research study was conducted on 62 carbapenem-resistant clinical isolates collected from November 2022 to February 2024. Ceftazidime–avibactam/aztreonam synergy against 55 carbapenemase producers [New Delhi metallo-beta-lactamase (NDM), imipenem-hydrolysing metallo-beta-lactamase (IMP), Verona Integron-encoded metallo-beta-lactamase (VIM) and oxacillinase-48 (OXA-48)] was determined using the disc diffusion method. Data analysis was performed by chi-square test. Ceftazidime–avibactam/aztreonam synergy was identified against 25 (64.1%) out of 39 isolates exhibiting the NDM gene, seven (77.8%) out of nine isolates that were co-producers of NDM and OXA-48 genes, two (50%) out of four isolates co-producing NDM and VIM carbapenemase genes and a single isolate (33.3%) out of three isolates with NDM, VIM and OXA-48 genes. A wide zone of 3–23 mm diameter was observed for *Enterobacterales* and 6–7 mm for *P. aeruginosa* with ceftazidime–avibactam/aztreonam in relative to ceftazidime–avibactam and aztreonam discs when tested alone. More than 30% of isolates showed a statistically significant difference in zone diameter for the ceftazidime–avibactam/aztreonam combination (*P*<0.05), when compared with the zone size for ceftazidime–avibactam and aztreonam discs when tested alone. The present study showed the *in vitro* effectiveness of the ceftazidime–avibactam/aztreonam combination against 63.6% of carbapenem-resistant isolates studied. The disc diffusion method requires less technical expertise, and the test result aids in identifying true clinical synergy by observing the widening of the zone diameter that exceeds the aztreonam susceptibility breakpoint.

## Data Summary

The authors confirm that all supporting data and protocols are provided within the manuscript.

## Introduction

Carbapenem-resistant Gram-negatives pose a significant public health threat due to the presence, acquisition or production of carbapenemases, specifically metallo-beta-lactamases (MBLs). Non-carbapenemase-producing carbapenem-resistant strains generate other resistance mechanisms influenced by outer membrane porin mutation or an efflux pump, or both, with the production of extended-spectrum beta-lactamase (ESBL) or AmpC beta-lactamase [1]. Plasmid-mediated carbapenemases are Ambler class A members [*Serratia marcescens* enzyme (SME), not metallo enzyme carbapenemase (NMC), *Klebsiella pneumoniae* carbapenemase (KPC) and Guiana extended spectrum (GES)], class B [imipenem-hydrolysing MBL (IMP), Verona Integron-encoded MBL (VIM), German imipenemase (GIM), Sao Paulo MBL (SPM), Seoul imipenemase (SIM) and New Delhi MBL (NDM-1)] and class D [oxacillinase-48 (OXA-48)] beta-lactamases [2]. Carbapenemase-producing *Enterobacterales* or non-fermenting bacilli infections are associated with higher mortality rates, varying between 40% and 50% [3].

Class B MBLs show varying degrees of hydrolysis against all beta-lactams, excluding monobactams like aztreonam [1]. Among the nine MBL types reported worldwide, IMP, NDM and VIM are the most notable genes in Africa, Asia, European nations and the Americas [4]. These MBLs (IMP, NDM and VIM) can deactivate nearly all beta-lactam drugs utilized clinically, with the exception of monobactams.

Ceftazidime–avibactam consists of a third-generation cephalosporin combined with a novel beta-lactamase inhibitor [5]. Avibactam inhibits class A beta-lactamases, chromosomal class C (AmpC), plasmid class C and some class D (OXA-48) beta-lactamases; it is not active against MBLs. Ceftazidime–avibactam shows significant activity against *K. pneumoniae* carbapenemase and OXA-48-like carbapenemases, ESBLs and AmpC, along with effectiveness towards non-carbapenemase-producing pathogens like carbapenem-resistant *Enterobacterales* [6]. Aztreonam is stable in the presence of MBL enzymes; therefore, the combination of ceftazidime–avibactam with aztreonam may have clinical utility in treating infections caused by MBL-producing *Enterobacterales*. In recent years, there has been an increase in reports of *Enterobacterales* harbouring multiple enzymes, particularly NDM and OXA-48. It is crucial to understand the effective antimicrobials against these isolates to improve clinical outcomes. The synergistic activity of the combination of ceftazidime–avibactam and aztreonam against co-producers of NDM and OXA-48 in *K. pneumoniae* has been observed, with clinical and microbiological recovery in patients [7].

There is no recommended standard method for testing ceftazidime–avibactam/aztreonam synergy. Disc stacking, broth disc elution, gradient strip stacking and strip crossing have been introduced in various studies to detect synergy [8,10]. The key principle for these testing methods for ceftazidime–avibactam/aztreonam synergy is based on restoration of the aztreonam zone size or aztreonam MIC when tested with a constant concentration of avibactam (4 µg ml^−1^) by E-strip disc diffusion or combined disc test [11]. These methods also require precise placement of E-strips or discs at a predetermined distance on the agar surface. The E-strip-based synergy testing is relatively expensive to perform in resource-limited settings.

The disk diffusion method offers many advantages over E-strip-based methods due to its cost-effectiveness and ease of use in performing and interpreting results. Disk replacement and double-disc synergy tests are the most studied disc diffusion methods to detect ceftazidime–avibactam/aztreonam synergy. Inaccurate spacing of discs and incorrect interpretation of characteristic synergy zones can lead to erroneous results. The disk replacement method is time-consuming and requires preincubation time for 1 h before placing the aztreonam disc, followed by incubation for 16–18 h at 37 °C [10]. The present study evaluated a simple disc diffusion method that does not require a preincubation time, making it less time-consuming and technically less demanding to detect the *in vitro* efficacy of the ceftazidime–avibactam/aztreonam combination against NDM-, VIM- and OXA-48-producing carbapenemase co-producers.

## Aims and objectives

1. To evaluate the *in vitro* activity of ceftazidime–avibactam/aztreonam against carbapenem-resistant clinical isolates.
2. To evaluate the synergistic activity of ceftazidime–avibactam/aztreonam against multiple carbapenemase producers.

## Methods

A cross-sectional study was conducted on 62 carbapenem-resistant clinical isolates collected from November 2022 to February 2024. Isolates were obtained from various sources, including urine, pus, blood, respiratory specimens and body fluids. Samples were collected under strict aseptic conditions from outpatient and inpatient units and were transported to the Central Laboratory at Vinayaka Mission’s Kirupananda Variyar Medical College and Hospitals, Salem, for further processing. Phenotypic characterization of the isolates by Vitek 2 was performed at the Microbiology Laboratory, Microbe World, Salem.

Samples were inoculated onto blood agar and MacConkey agar and incubated overnight at 37 °C. Gram-negative bacterial isolates were identified by microscopic morphology, colony morphology examination and biochemical reactions. Isolates were subjected to the catalase test, oxidase test, mannitol motility medium, triple sugar iron agar test, indole test, citrate utilization test and urea hydrolysis test for further identification and speciation. Antimicrobial susceptibility testing using the Kirby–Bauer disc diffusion method was performed with the following discs (HiMedia Laboratories Pvt. Ltd., Mumbai, India): amikacin (30 µg), amoxyclav (20/10 µg), aztreonam (30 µg), cefoperazone/sulbactam (75/30 µg), cefotaxime (30 µg), cefepime (30 µg), ceftazidime (30 µg), ceftazidime–avibactam (30/20 µg), ciprofloxacin (5 µg), co-trimoxazole (25 µg), gentamicin (10 µg), imipenem (10 µg), meropenem (10 µg) and piperacillin/tazobactam (100/10 µg). *Escherichia coli* ATCC 25922 and *Pseudomonas aeruginosa* ATCC 27853 were used as control strains for susceptibility testing.

The zone of inhibition was measured in millimetres using Clinical Laboratory Standards Institute (CLSI) interpretive criteria. A ceftazidime–avibactam zone size of ≥21 mm was considered susceptible, while ≤20 mm was considered resistant. For aztreonam, an inhibition zone of ≥21 mm was regarded as susceptible, 18–20 mm as intermediate and ≤17 mm as resistant for *Enterobacterales*. A zone diameter of ≥22 mm was considered susceptible, 16–21 mm as intermediate and ≤15 mm as resistant for *P. aeruginosa* isolates. An inhibition zone of ≤19 mm for imipenem and meropenem was considered resistant for *Enterobacteriaceae,* while a zone diameter of ≤15 mm was considered resistant for *Pseudomonas* strains [12,13]. Sixty-two isolates that exhibited resistance to either imipenem or meropenem, or both, were characterized phenotypically using the Vitek 2 automated system. A turbidometrically controlled suspension of organisms was used to inoculate Vitek 2 Identification (ID)/antimicrobial-susceptibility testing (AST) Gram-negative cards (AST-N405).

The MIC for the following antibiotics, viz imipenem, meropenem, amikacin, gentamicin, piperacillin/tazobactam, amoxycillin–clavulanate, cefepime, ciprofloxacin, co-trimoxazole and tigecycline, was determined using Vitek 2 ID/AST cards.

### Ceftazidime–avibactam/aztreonam synergy testing

Susceptibility to individual discs (ceftazidime–avibactam and aztreonam disc) was detected by placing the individual disc on a culture suspension on one half of Muller–Hinton agar plate. To detect synergy, disc-based susceptibility testing was performed by placing an aztreonam (30 µg) disc on ceftazidime–avibactam (30/20 µg) on the other half of the petri plate, ensuring that one edge of the aztreonam disc was embedded in the inoculated media, or by placing the ceftazidime–avibactam disc on the aztreonam disc, ensuring that the edge of the ceftazidime–avibactam disc was embedded in the inoculated media, and incubated at 37 °C overnight. The zone of inhibition diameter for the ceftazidime–avibactam/aztreonam combination was analysed as per CLSI guidelines [12].

If a particular isolate was observed to be resistant to both aztreonam and ceftazidime–avibactam when tested alone, but showed a zone of inhibition in the susceptible range for aztreonam when combined with ceftazidime–avibactam, it was considered as synergism-positive.

If a particular strain was observed to be resistant to aztreonam and ceftazidime–avibactam when tested alone and showed a zone of inhibition in the resistant range for aztreonam when combined with ceftazidime–avibactam, it was considered as synergism-negative.

If a particular strain was found to be susceptible to aztreonam alone and/or ceftazidime–avibactam alone, it was taken as synergism-negative [13].

The confirmation of carbapenem-resistant isolates at the genotypic level was done using the Hi-PCR Carbapenemase Gene (Multiplex) Probe PCR Kit (HiMedia Laboratories Pvt. Ltd., Mumbai, India). To extract and purify DNA, the HiPurA Bacterial Genomic DNA Purification Kit (HiMedia Laboratories Pvt. Ltd., Mumbai, India) was used. Target genes, including KPC, IMP, NDM, VIM, OXA-51, OXA-23, OXA-48 and OXA-58, were identified by real-time PCR: initial denaturation at 95 °C for 10 min, followed by a denaturation cycle at 95 °C for 5 s, with subsequent annealing and extension cycle at 60 °C for 1 min. The threshold cycle (Ct) value of ≤40 was interpreted as positive for carbapenem-resistant genes. Lack of amplification curve in the target genes channel was taken as negative. Analysis of data was performed by the chi-square test; a *P*-value of <0.05 was taken as statistically significant.

## Results

Out of 62 isolates with carbapenem resistance, 55 (88.7%) were phenotypically confirmed by Vitek 2. Among the 55 Gram-negative bacteria exhibiting carbapenem resistance, 23 were *E. coli,* 14 were *K. pneumoniae*, seven were *P. aeruginosa*, four were *Enterobacter cloacae*, four were *Acinetobacter baumannii*, and one isolate each was *Proteus mirabilis*, *Pseudomonas alcaligenes* and *Pseudomonas fluorescens*.

All isolates (100%) showed non-susceptibility to ceftazidime (MIC: ≥64 µg ml^−1^) and cefepime (MIC: 16–64 µg ml^−1^). Fifty-four (98.2%) out of 55 isolates exhibited non-susceptibility to piperacillin–tazobactam (MIC: ≥128 µg ml^−1^) and ciprofloxacin (MIC: ≥4 µg ml^−1^). Twenty-five (45.5%) isolates revealed resistance to amikacin (MIC: ≥64 µg ml^−1^), and 37 (67.3%) isolates showed non-susceptibility to gentamicin (MIC: ≥16 µg ml^−1^). Fifty-four (98.2%) isolates exhibited a MIC ranging from 4 to 64 µg ml^−1^ for imipenem, while 55 (100%) isolates demonstrated a MIC of 4–64 µg ml^−1^ for meropenem.

**Fig. 1.**
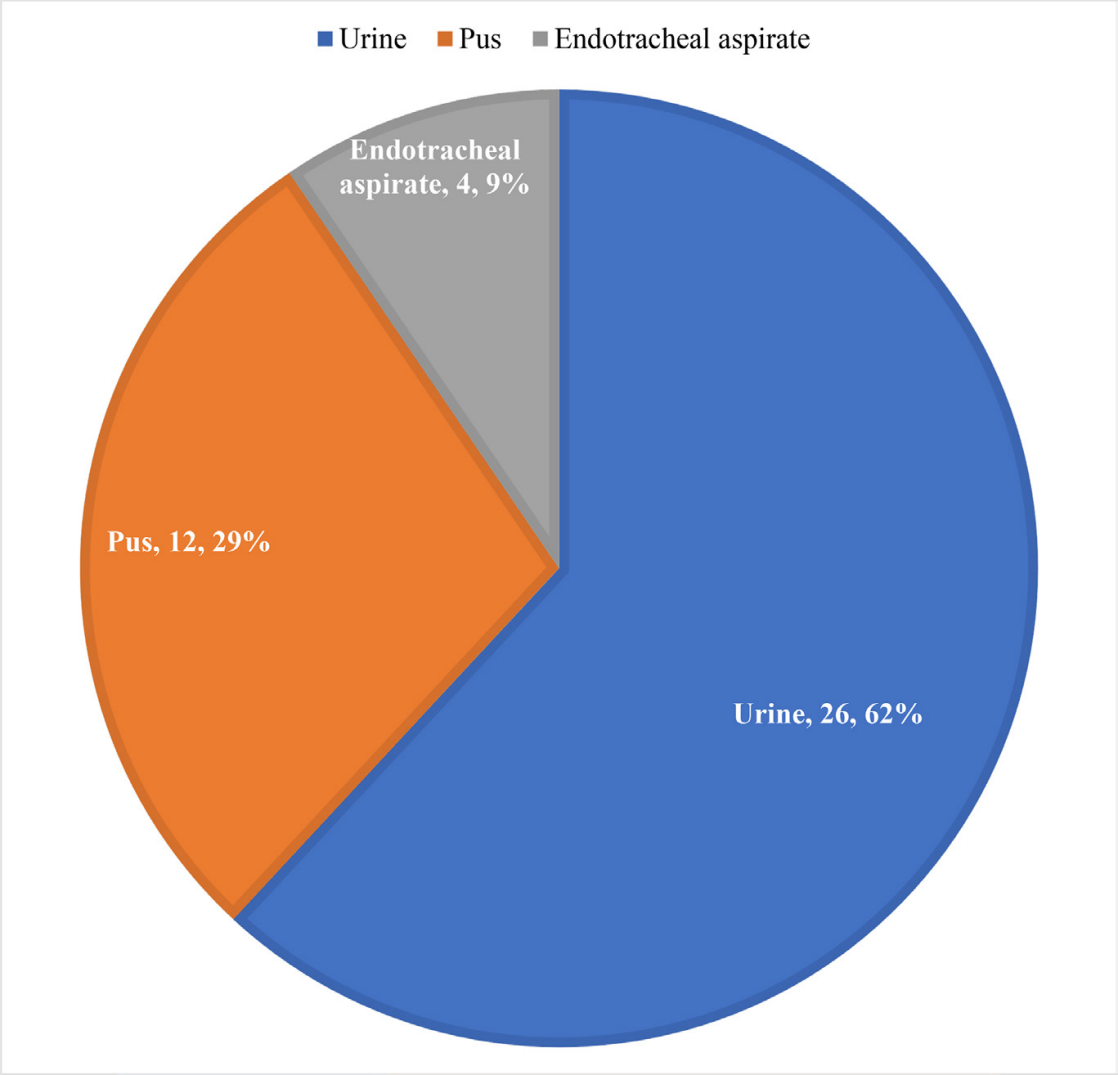
Percentage distribution of carbapenem-resistant *Enterobacterales* from clinical samples.

Carbapenem resistance was observed among 42 *Enterobacterales.* Out of 13 carbapenem-resistant non-fermenters studied, five (38.5%) isolates were from urine, three (23.1%) from pus samples, two (15.4%) from bronchoalveolar lavage and three (23.1%) from endotracheal aspirates (Fig. 1).

**Fig. 2.**
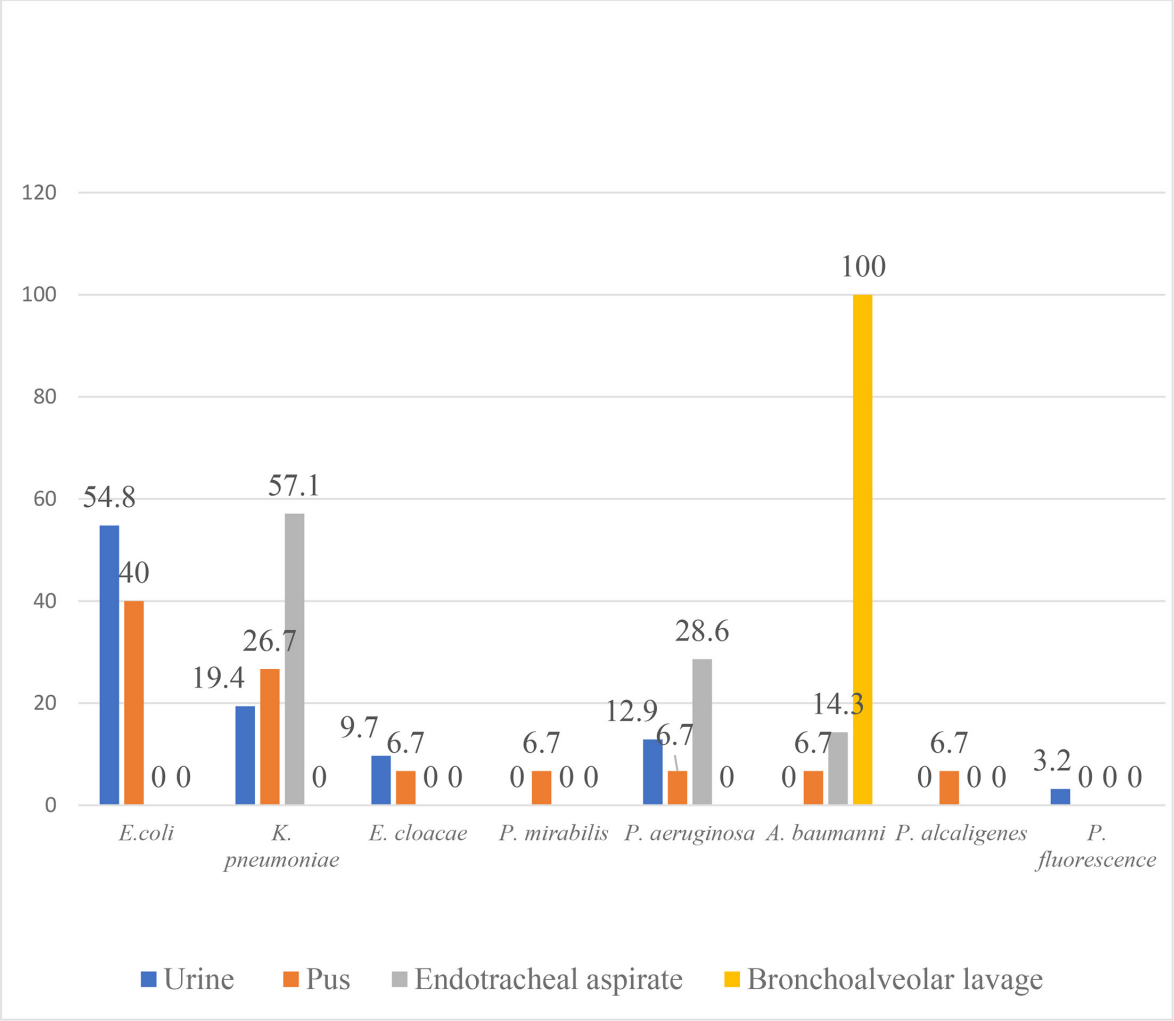
Sample-wise distribution of carbapenem-resistant *Enterobacterales* and non-fermenters*.*

Seventeen carbapenem-resistant *E. coli* isolates were from urine samples, and six were from pus samples. Six carbapenem-resistant *K. pneumoniae* isolates were from urine samples, four from pus samples and four from endotracheal aspirates. Four carbapenem-resistant *P. aeruginosa* isolates were from urine samples, one from pus and two from endotracheal aspirates (Fig. 2).

**Fig. 3.**
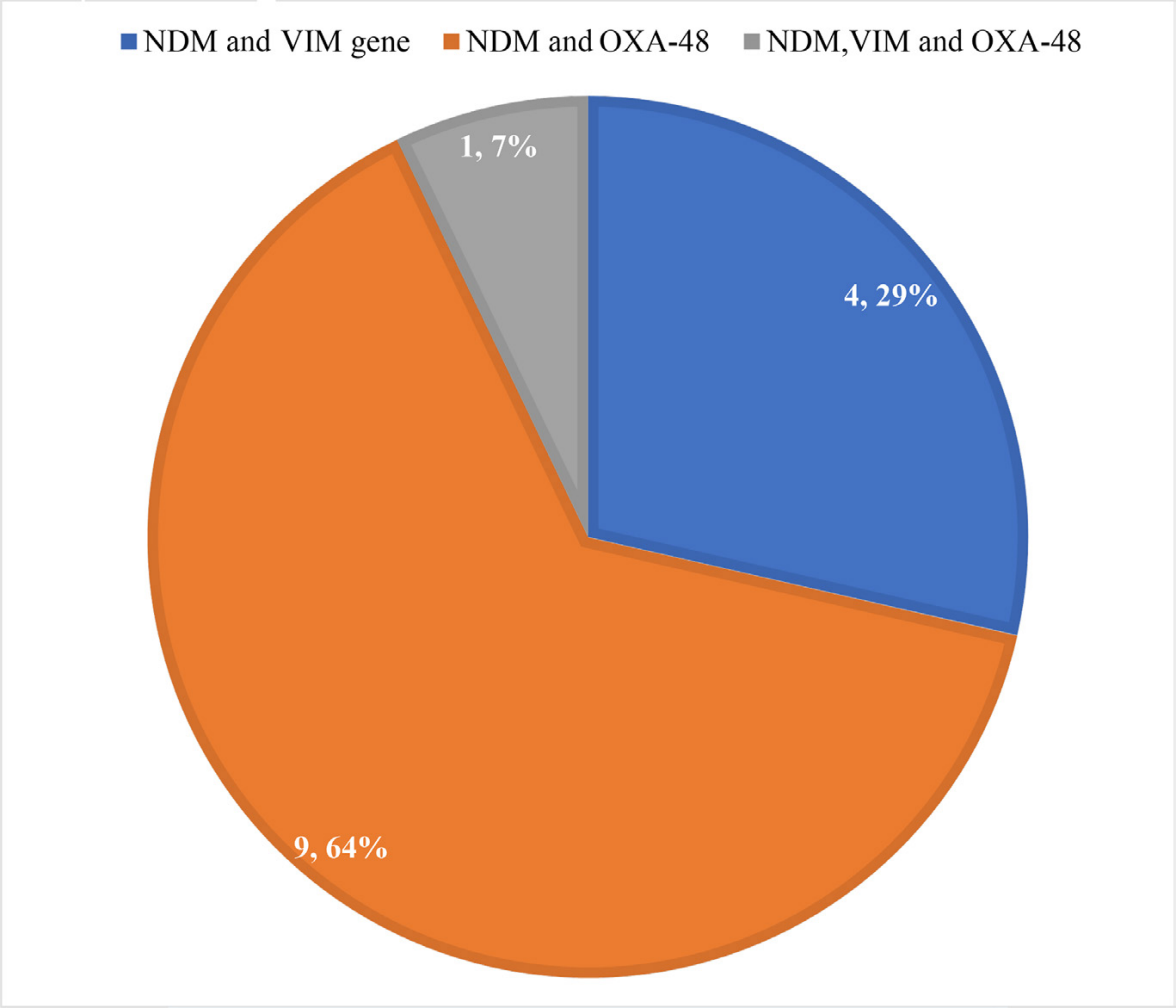
Distribution of multiple carbapenem-resistant genes in clinical isolates.

The presence of multiple carbapenem-resistant genes was observed in 14/55 (25.5%) isolates (Fig. 3). Thirty-nine (70.9%) isolates confirmed the presence of the NDM gene alone. A single isolate (1.8%) each indicated the presence of the VIM gene alone and the IMP gene alone.

Combined production of NDM and OXA-48 genes was observed among three *E. coli* isolates, one *K. pneumoniae* isolate obtained from a urine sample, four *K*. *pneumoniae* isolates from endotracheal aspirates and one *P. mirabilis* isolate from a pus sample. A single isolate each of *E. coli*, *K. pneumoniae* and *P. fluorescens* from urine samples, as well as a single isolate of *E. coli* from pus, were co-producers of NDM and VIM genes. A single isolate of *K. pneumoniae* from a urine sample showed the existence of NDM, VIM and OXA-48 genes. A single isolate of *P. aeruginosa* from a pus sample showed the presence of the VIM gene alone.

**Fig. 4.**
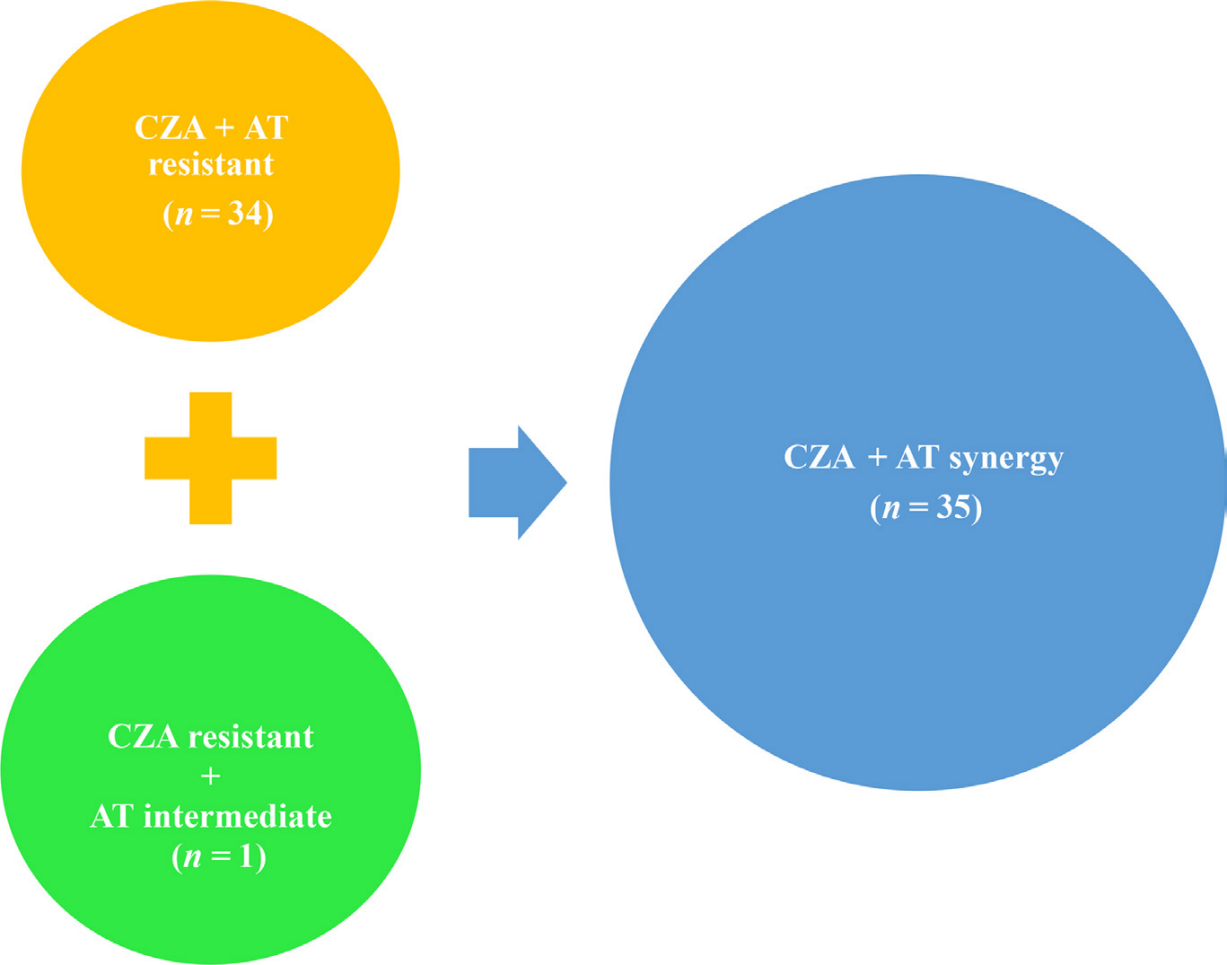
CZA and AT resistance profile of isolates with CZA+AT combination synergism. AT, aztreonam; CZA, ceftazidime–avibactam.

Out of 55 carbapenem-resistant isolates studied, 35 (63.6%) showed synergism to the ceftazidime–avibactam/aztreonam combination. Out of these 35 isolates, 34 were resistant to both ceftazidime–avibactam and aztreonam discs when tested individually (Fig. 4).

**Fig. 5.**
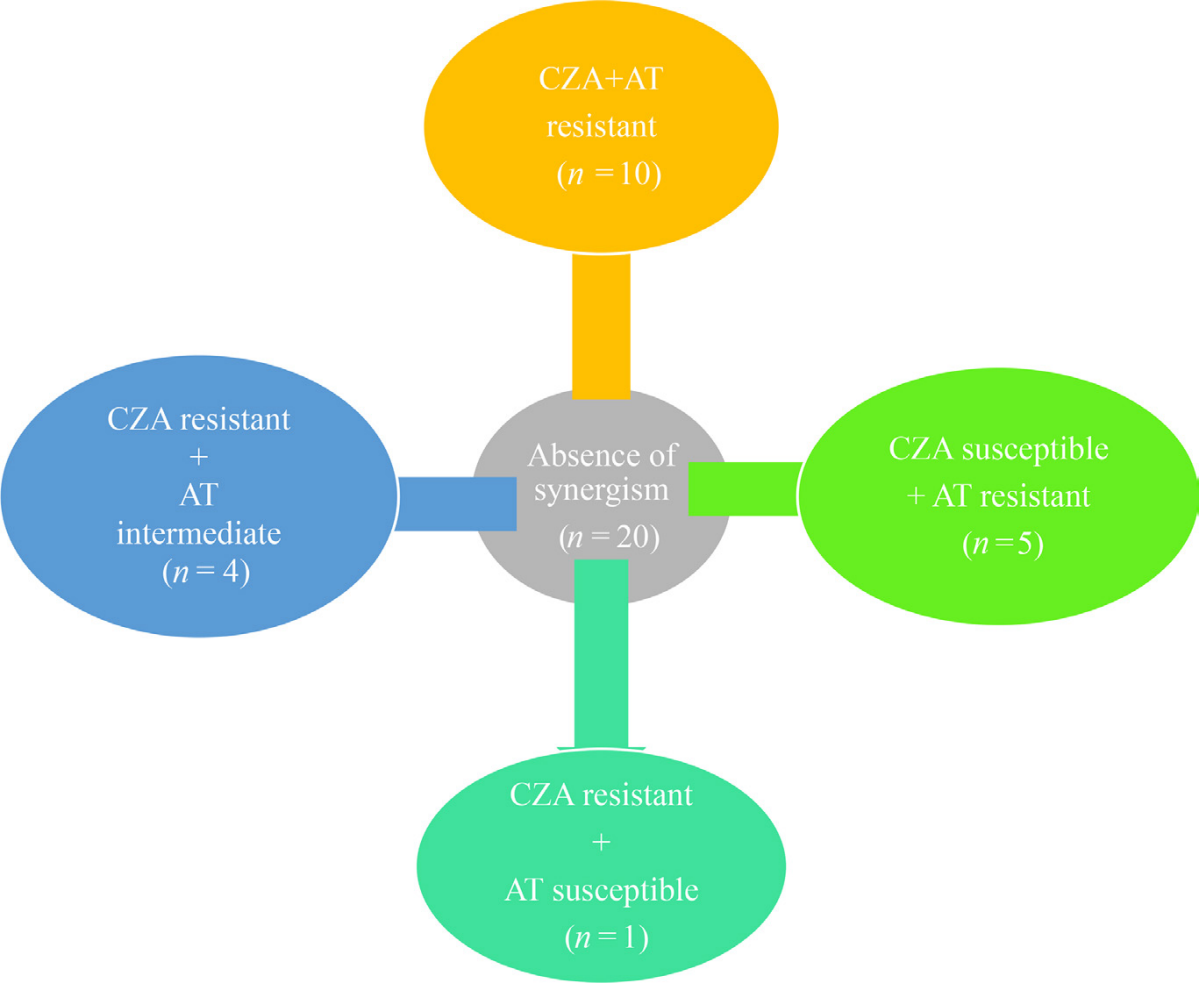
CZA and AT resistance profile of isolates with the absence of CZA+AT combination synergism. AT, aztreonam; CZA, ceftazidime–avibactam.

Twenty (36.4%) out of 55 isolates showed an absence of synergism for the ceftazidime–avibactam/aztreonam combination. Out of these 20 isolates, ten were resistant to both ceftazidime–avibactam and aztreonam discs when tested individually (Fig. 5).

Out of 42 carbapenem-resistant *Enterobacterales*, 37 (88.1%) isolates exhibited resistance to ceftazidime–avibactam, and 41 (97.6%) isolates showed resistance to aztreonam. Two *E. coli* isolates and a single isolate each of *K. pneumoniae* and *P. mirabilis* showed intermediate resistance to aztreonam. Thirty-two (76.2%) out of 42 isolates exhibited synergy to the ceftazidime–avibactam/aztreonam combination by disc diffusion (Table 1).

**Table 1.** Distribution of carbapenem-resistant isolates from clinical samples showing ceftazidime–avibactam/aztreonam synergy

S. No.	Sample	Organism	Zone diameter in millimetre				***P*-value**
			CZA (R)	AT (R)	CZA/AT zone interpretation		
1	Pus (*n*=10)	*E. coli*	6 mm	11 mm	23 mm	Susceptible	**0.00722**
		*E. coli*	14 mm	6 mm	17 mm	Resistant	0.10207
		*E. coli*	14 mm	6 mm	17 mm	Resistant	0.10207
		*E. coli*	12 mm	10 mm	19 mm	Intermediate	0.25111
		*E. coli*	12 mm	13 mm	22 mm	Susceptible	0.18155
		*K. pneumoniae*	6 mm	17 mm	29 mm	Susceptible	**0.00099**
		*K. pneumoniae*	12 mm	6 mm	22 mm	Susceptible	**0.01486**
		*K. pneumoniae*	12 mm	6 mm	19 mm	Intermediate	0.05588
		*E. cloacae*	15 mm	16 mm	22 mm	Susceptible	0.51007
		*P. mirabilis*	14 mm	19 mm	23 mm	Susceptible	0.51007
2	Urine (*n*=22)	*E. coli*	14 mm	16 mm	19 mm	Intermediate	0.78455
		*E. coli*	20 mm	6 mm	20 mm	Intermediate	**0.01772**
		*E. coli*	14 mm	6 mm	20 mm	Intermediate	**0.04041**
		*E. coli*	6 mm	6 mm	16 mm	Resistant	0.06081
		*E. coli*	16 mm	6 mm	20 mm	Intermediate	**0.02437**
		*E. coli*	16 mm	6 mm	21 mm	Susceptible	**0.02655**
		*E. coli*	11 mm	12 mm	13 mm	Resistant	0.92004
		*E. coli*	14 mm	6 mm	18 mm	Intermediate	0.08208
		*E. coli*	14 mm	6 mm	14 mm	Resistant	0.18048
		*E. coli*	14 mm	6 mm	17 mm	Resistant	0.10207
		*E. coli*	15 mm	6 mm	15 mm	Resistant	0.1054
		*E. coli*	10 mm	10 mm	15 mm	Resistant	0.55593
		*K. pneumoniae*	12 mm	6 mm	22 mm	Susceptible	0.30119
		*K. pneumoniae*	12 mm	6 mm	25 mm	Susceptible	**0.00315**
		*K. pneumoniae*	12 mm	11 mm	18 mm	Intermediate	0.39403
		*K. pneumoniae*	12 mm	12 mm	25 mm	Susceptible	0.056
		*P. aeruginosa*	6 mm	6 mm	13 mm	Resistant	0.24935
		*P. aeruginosa*	6 mm	6 mm	13 mm	Resistant	0.24935
		*P. aeruginosa*	6 mm	6 mm	14 mm	Resistant	0.11779
		*E. cloacae*	14 mm	11 mm	27 mm	Susceptible	**0.02806**
		*E. cloacae*	6 mm	9 mm	14 mm	Resistant	0.25924
		*E. cloacae*	10 mm	6 mm	19 mm	Intermediate	**0.03527**
3	Endotracheal aspirate (*n*=3)	*K. pneumoniae*	6 mm	6 mm	29 mm	Susceptible	**<0.00001**
		*K. pneumoniae*	10 mm	12 mm	14 mm	Resistant	0.71653
		*K. pneumoniae*	6 mm	6 mm	29 mm	Susceptible	**<0.00001**

Among 13 non-fermenters, 11 (84.6%) isolates displayed resistance to ceftazidime–avibactam and aztreonam when tested separately. One isolate (7.7%) showed ceftazidime–avibactam susceptibility and aztreonam disc resistance when tested separately. One isolate (7.7%) showed resistance to ceftazidime–avibactam and susceptibility to aztreonam disc when tested alone.

A statistically significant difference in zone diameter for the ceftazidime–avibactam/aztreonam combination (*P*<0.05) was observed in 29.4% *E. coli* isolates and 50% each of *K. pneumoniae* and *E. cloacae* isolates.

Synergy between ceftazidime–avibactam and aztreonam was observed among 25 out of 39 isolates with the NDM gene, including ten isolates with multiple carbapenemase enzymes (seven co-producers of the NDM and OXA-48 genes, two co-producers of NDM and the VIM carbapenemase gene and a single isolate with NDM, VIM and OXA-48 genes).

Synergy between ceftazidime–avibactam and aztreonam was also observed among *E. coli* isolates with multiple carbapenemase enzymes (three co-producers of NDM and OXA-48 genes, two co-producers of NDM and VIM genes) and 12 isolates with the NDM genes. Six *E. coli* isolates with the NDM carbapenemase gene showed an absence of ceftazidime–avibactam/aztreonam synergism (Table 2).

**Table 2.** Evaluation of ceftazidime–avibactam/aztreonam combination synergism against isolates with multiple carbapenemase enzymes

Isolate	Carbapenemase enzyme detected	Synergism	No synergism	*P*-value*
*E. coli* (*n*=18)	NDM alone	12 (66.67%)	6 (33.33%)	0.1573
*E. coli* (*n*=3)	NDM and OXA-48	3 (100%)	0 (0%)	–
*E. coli* (*n*=2)	NDM and VIM	2 (100%)	0 (0%)	–
*K. pneumoniae* (*n*=7)	NDM alone	6 (85.71%)	1 (14.29%)	0.1573
*K. pneumoniae* (*n*=3)	NDM and OXA-48	3 (100%)	0 (0%)	–
*K. pneumoniae* (*n*=1)	NDM, VIM and OXA-48	1 (100%)	0 (0%)	–
*K. pneumoniae* (*n*=3)	NDM and VIM	0 (0%)	3 (100%)	–
*E. cloacae* (*n*=4)	NDM alone	4 (100%)	0 (0%)	–
*P. mirabilis* (*n*=1)	NDM and OXA-48	1 (100%)	0 (0%)	–
*P. aeruginosa* (*n*=5)	NDM alone	3 (60.00%)	2 (40.00%)	0.65472
*P. aeruginosa* (*n*=1)	VIM alone	0 (0%)	1 (100%)	–
*P. aeruginosa* (*n*=1)	IMP alone	0 (0%)	1 (100%)	–
*P. alcaligenes* (*n*=1)	NDM alone	0 (0%)	1 (100%)	–
*P. fluorescens *(*n*=1)	NDM and VIM	0 (0%)	1 (100%)	–
*A. baumannii* (*n*=4)	NDM alone	0 (0%)	4 (100%)	–

No statistically significant association was found among isolates harbouring carbapenemase enzymes and synergism towards the ceftazidime–avibactam/aztreonam combination.

* P value could not be calculated for the rows with dashes

Synergy to the ceftazidime–avibactam/aztreonam combination was observed among *K. pneumoniae* with multiple carbapenemase enzymes (three co-producers of the NDM and OXA-48 genes and one isolate with NDM, VIM and OXA-48 genes), and six isolates with the NDM gene.

Ceftazidime–avibactam/aztreonam synergy was also observed among three isolates of *P. aeruginosa* with the NDM carbapenemase gene. Absence of synergy was noted in a single isolate each of *P. aeruginosa* with the VIM carbapenemase gene alone and the IMP gene alone, as well as in two isolates with the NDM gene alone.

Out of 35 isolates with ceftazidime–avibactam/aztreonam combination synergism, 18 isolates (seven *E. coli*, four *P. aeruginosa*, three *K*. *pneumoniae*, three *E. cloacae* and one *P*. *mirabilis*) were randomly selected and subjected to the disc replacement method and double-disc synergy testing. The results of the disc diffusion method were found to be concordant with the disc replacement and double-disc synergy tests.

## Discussion

Carbapenem-resistant *Enterobacterales* are increasing worldwide and pose a great threat to global public health, particularly due to MBL producers. The therapeutic options for MBL producers vary depending on the susceptibility profile of individual drugs, resorting to individual drugs such as polymyxins, aminoglycosides, tetracyclines and fosfomycin, in the absence of clear therapeutic consensus and while facing numerous safety concerns [14]. The optimal therapy for MBL-producing *Enterobacterales* or *P. aeruginosa* remains indistinct, being correlated with insufficient clinical experience and a limited range of therapeutic alternatives. The introduction of ceftazidime–avibactam combination has made MBL treatment possible with beta-lactam drugs, overcoming the toxic effects (renal injury) and poor lung penetration associated with the use of polymyxins [15].

The predominance of carbapenemase enzymes varies in India and the western world, depending on the enzyme epidemiology in the region. The carbapenemase groups most commonly encountered in India include NDM and OXA-48-like groups [16]. In the present study, we evaluated the predominant carbapenemase groups in our clinical setting and the effectiveness of the ceftazidime–avibactam/aztreonam combination against the identified carbapenemase groups. NDM (96.3%) was the predominant carbapenemase producer detected among clinical isolates. The presence of diverse carbapenem-resistant genes was found in 25.5% of the isolates. Prayag *et al.* [17] reported the predominance of NDM, OXA-48 and KPC enzymes among *Enterobacterales* in India. Haji *et al*. [1] reported NDM and OXA-48 as the predominant carbapenemases in their study, with 96% of the isolates possessing one or more carbapenemase genes. Klein *et al*. [18] reported that 95% of *P*. *aeruginosa* isolates had VIM as the common MBL group. Adam *et al.* [19] reported VIM and IMP as the predominant genes among Gram-negative clinical isolates. Additionally, 25.8% of uropathogenic *K. pneumoniae* isolates harboured multiple carbapenemase genes, as reported by Urmi *et al.* [4]. Mohamed *et al.* [20] indicated that NDM was the most frequently detected gene among *K. pneumoniae* isolates from ventilator-associated pneumonia. Studies on carbapenem-resistant clinical isolates highlight the predominance of NDM and OXA-48 among *Enterobacterales*, with multiple carbapenemase genes detected in *K. pneumoniae* and a significant rate of VIM enzyme in *P. aeruginosa*.

Disk-based methods [16,21, 22] recommended for detecting ceftazidime–avibactam/aztreonam synergism among MBL producers are labour-intensive, expensive and require a preincubation time ranging from 10 min to 1 h before placing aztreonam over the ceftazidime–avibactam disc. We employed a simple disc diffusion method that does not require preincubation time and is easy to interpret regarding zone diameter; therefore, it could be adopted in low-resource settings. Disk-based methods [1,21,23] described earlier have detected ceftazidime–avibactam/aztreonam synergism among 70–80% of NDM isolates. In the present study, 35 (63.6%) NDM isolates showed synergism to the combination drug by disc diffusion. Using the disc diffusion method, Sreenivasan *et al*. [22] documented *Enterobacterales* with an extended zone size of 4–16 mm for the ceftazidime–avibactam/aztreonam combination when compared with the individual zone sizes of ceftazidime–avibactam and aztreonam. In the present study, we observed an increased zone size of 3–23 mm for *Enterobacterales* and 6–7 mm for *P. aeruginosa* with the ceftazidime–avibactam/aztreonam combination.

Indian Council of Medical Research guidelines recommend the use of ceftazidime–avibactam/aztreonam combination against NDM-producing *Enterobacterales* [24]. In our study, 64.1% isolates with the NDM enzyme solely tested positive for synergy with the ceftazidime–avibactam/aztreonam combination, including 12 *E. coli*, six *K*. *pneumoniae*, four *E. cloacae* and three *P*. *aeruginosa*. Synergism was also observed against *E. coli* that co-produced both NDM and OXA-48, *E. coli* with NDM and VIM, as well as *K. pneumoniae* isolates with NDM and OXA-48, *K. pneumoniae* with NDM, VIM and OXA-48, and *P. mirabilis* with NDM and OXA-48.

The co-production of MBLs along with serine carbapenemases and ESBLs is common in *Enterobacterales*, which ultimately affects the choice of antibiotic and clinical outcomes. Biagi *et al*. [25] reported ceftazidime–avibactam/aztreonam synergy among 87.5% of clinical *Enterobacteriaceae* isolates that were co-producers of NDM with at least one serine beta-lactamase. Romanelli *et al*. [26] have demonstrated the synergistic effect of the ceftazidime–avibactam/aztreonam combination against co-producers of KPC and NDM. Sempere *et al*. [27] observed a synergistic effect (84%) against VIM-producing *Enterobacterales* and *P. aeruginosa,* and synergism correlated with clinical success among VIM *P. aeruginosa* isolates. The synergistic effect in inhibiting or killing bacteria against NDM-, IMP-, KPC+IMP- and KPC+NDM-producing strains was documented in a study by Lu *et al*. [28]. During this study period, serine beta-lactamases were not isolated from the clinical samples; hence, the *in vitro* synergism against serine beta-lactamases could not be demonstrated by this method in our study.

In our study, a statistically significant difference in zone diameter for the ceftazidime–avibactam/aztreonam combination (*P*<0.05) was observed in 34.3% isolates when compared with the zone size for ceftazidime–avibactam and aztreonam discs when tested alone. No statistically significant association was found among isolates harbouring carbapenemase enzymes and synergism towards the ceftazidime–avibactam/aztreonam combination.

Our study showed ceftazidime–avibactam/aztreonam effectiveness against co-producers of NDM, VIM and OXA-48. Prior to using this combination drug in patients, it is important to detect the *in vitro* synergy of aztreonam and ceftazidime–avibactam to assess clinical success. The ceftazidime–avibactam/aztreonam offers a therapeutic benefit compared with other active antibiotics for bloodstream infection due to MBL producers and pan-drug-resistant *K. pneumoniae* infection [29,30]. An Indian study demonstrated an 82.35% clinical cure rate in patients on combination therapy with ceftazidime–avibactam and polymyxin/tigecycline/fosfomycin [31].

In our study, a reference standard method for synergy testing was not included; therefore, the study result cannot be compared with a reference method. However, 18 out of 35 isolates that showed synergy using this method were randomly selected and compared with disc replacement and double-disc synergy testing, yielding comparable results. To evaluate the reproducibility of the test result within the laboratory, study isolates need to be tested in replicates. In low-resource settings, a simple disc diffusion method aids in screening for ceftazidime–avibactam/aztreonam synergy alongside routine antimicrobial susceptibility testing.

## Limitation of the study

The predominant carbapenemase co-producers in our study harboured NDM plus OXA-48 enzymes and NDM plus VIM. Due to the low number of isolates with the IMP gene, the *in vitro* efficacy of ceftazidime–avibactam/aztreonam against IMP isolates cannot be accurately evaluated. In our study, co-production of beta-lactamases was not found in *P. aeruginosa* isolates; hence, synergism was evaluated only against NDM-producing *P. aeruginosa*.

## Conclusion

The significance of combination regimens in the treatment of carbapenem-resistant clinical isolates is a matter of long-standing debate. Our study showed the efficacy of ceftazidime–avibactam/aztreonam against NDM, VIM and OXA-48 co-producers among *Enterobacterales* and *P. aeruginosa* possessing the NDM gene. The disc diffusion method requires less technical expertise to implement in routine laboratories, facilitating the evaluation of *in vitro* ceftazidime–avibactam/aztreonam synergy alongside routine antimicrobial susceptibility testing to assess clinical success.

## Abbreviations

AST: antimicrobial-susceptibility testing
AT: aztreonam
CLSI: Clinical Laboratory Standards Institute
CZA: ceftazidime–avibactam
ESBLs: extended-spectrum beta-lactamases
GES: Guiana extended spectrum
GIM: German imipenemase
ID: Identification
IMP: imipenem-hydrolysing metallo-beta-lactamase
KPC: *Klebsiella pneumoniae* carbapenemase
MBL: metallo-beta-lactamase
NDM: New Delhi metallo-beta-lactamase
NMC: not metallo enzyme carbapenemase
OXA-48: oxacillinase-48
SIM: Seoul imipenemase
SME: *Serratia marcescens* enzyme
SPM: Sao Paulo metallo-beta-lactamase
VIM: Verona Integron-encoded metallo-beta-lactamase
